# Dynamics of changing impacts of tropical Indo-Pacific variability on Indian and Australian rainfall

**DOI:** 10.1038/srep31767

**Published:** 2016-08-22

**Authors:** Ziguang Li, Wenju Cai, Xiaopei Lin

**Affiliations:** 1 Physical Oceanography Laboratory/CIMST, Ocean University of China and Qingdao National Laboratory for Marine Science and Technology, Qingdao 266100, China; 2 CSIRO Oceans and Atmosphere, Aspendale, VIC 3195, Australia

## Abstract

A positive Indian Ocean Dipole (IOD) and a warm phase of the El Niño-Southern Oscillation (ENSO) reduce rainfall over the Indian subcontinent and southern Australia. However, since the 1980s, El Niño’s influence has been decreasing, accompanied by a strengthening in the IOD’s influence on southern Australia but a reversal in the IOD’s influence on the Indian subcontinent. The dynamics are not fully understood. Here we show that a post-1980 weakening in the ENSO-IOD coherence plays a key role. During the pre-1980 high coherence, ENSO drives both the IOD and regional rainfall, and the IOD’s influence cannot manifest itself. During the post-1980 weak coherence, a positive IOD leads to increased Indian rainfall, offsetting the impact from El Niño. Likewise, the post-1980 weak ENSO-IOD coherence means that El Niño’s pathway for influencing southern Australia cannot fully operate, and as positive IOD becomes more independent and more frequent during this period, its influence on southern Australia rainfall strengthens. There is no evidence to support that greenhouse warming plays a part in these decadal fluctuations.

ENSO and the IOD are dominant modes of air-sea coupled interannual variability of the tropical Pacific and Indian Oceans^1^,^2^,^3^,^4^. They affect rainfall over tropical and extratropical regions^5^,^6^,^7^,^8^,^9^,^10^,^11^,^12^, including being associated with lower than normal rainfall over the Indian subcontinent and southern Australia in the boreal summer months (June-September, JJAS) during an El Niño or positive IOD developing year. In each region, JJAS rainfall accounts for more than half of the annual total^13^,^14^, making it crucially important for agriculture.

Year-to-year variations of rainfall in these two regions have been examined from the perspective of individual and combined impacts from ENSO and the IOD^9^,^12^,^15^,^16^,^17^,^18^. Indices of the two modes have been used in an attempt to improve the predictability of regional rainfall^19^,^20^. It is found that the impact from an El Niño and a positive IOD on the Indian summer monsoon rainfall (ISMR) can offset each other, whereas the impact of El Niño on southern Australia rainfall (SAR) can reinforce that of a positive IOD event. Although these teleconnections are triggered by the same source of anomalous diabatic heating, their impacts on the two regions are quite complex^16^,^18^,^21^.

Several studies have found that the ISMR-ENSO relationship has weakened since the 1980s^21^,^22^, but there is no consensus on the cause of the weakening. One suggestion is that the weakening may be attributed to greenhouse warming whereby warming of the Eurasian Continent increases the land-sea thermal contrast, leading to a strong monsoon^21^,^23^ and increased frequency of positive IOD events^24^. These factors may alter the contribution from ENSO. Other suggested causes for the weakening include intrinsic decadal variability of the monsoon^25^, stochastic nature of the monsoon^26^, and forcing from the Atlantic Oscillation^27^.

Motivated by the lack of a consensus, here we show that there is a similar change in the SAR-ENSO relationship since the 1980s and both changes are partly attributable to interdecadal variations in the coherence between ENSO and the IOD. Further, the Coupled Model Intercomparison Project Phase 5 (CMIP5) models^28^ show no changes in ENSO-IOD coherence, suggesting that the observed weakening can occur without global warming.

## Results

### Changing relationship between tropical variability and regional rainfall

**Figure 1:**
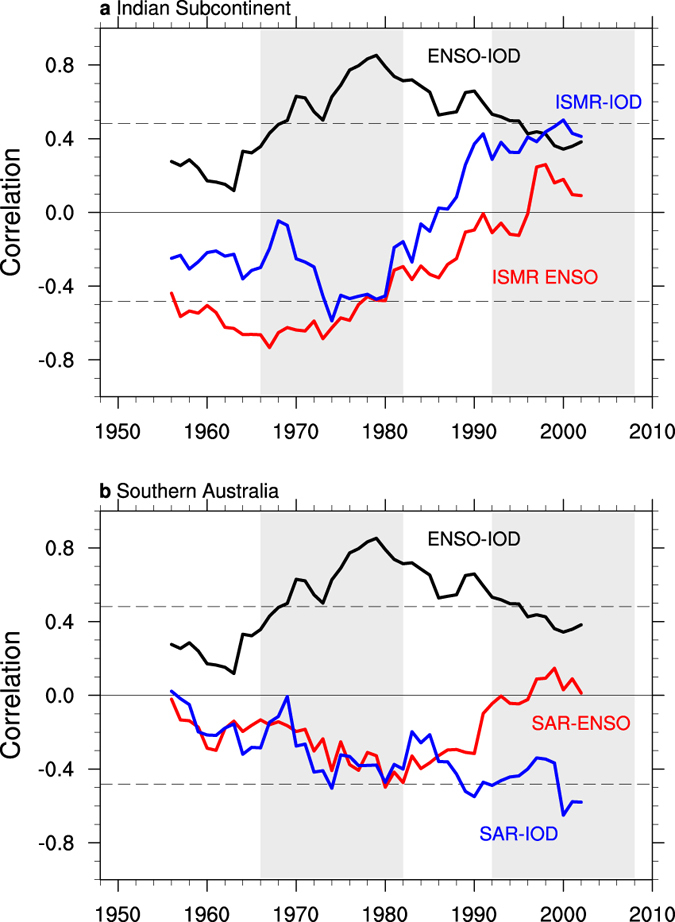
Sliding correlation between tropical variability and regional rainfall. (**a**) Sliding correlations between JJAS seasonal anomalies of the ISMR and ENSO (red line), the ISMR and the IOD (blue line), and ENSO and IOD (black line) on 17-year windows. Values are plotted at the centre of each 17-year period and statistical significance above the 95% confidence level by a two tailed Student’s *t*-test is achieved when the correlation is greater than 0.482 in amplitude, which indicated by dashed lines. Shaded boxes denote the sub-periods of 1966–1982 and 1992–2008, respectively. (**b**) As in (**a**), but for SAR. All plots were generated by NCL version 6.3.0 (http://dx.doi.org/10.5065/D6WD3XH5).

Over the Indian subcontinent, ENSO influences ISMR leading to lower than normal rainfall during an El Niño, and vice-versa during La Niña. However, the relationship fluctuated and weakened to a statistically insignificant level around 1980 and continued to weaken thereafter (Fig. 1a, red line), as reported by earlier studies^21^,^22^. Using observations and a model simulation, it has been suggested that without El Niño’s impact, a positive IOD event is associated with anomalously high rainfall, opposite to the impact of El Niño, thus modulating ENSO’s impact^15^,^16^. Indeed, accompanying the weak impact from ENSO, the IOD’s effect on ISMR also weakened to the extent that the ISMR actually increased during a positive IOD and the ISMR-IOD relationship is significantly positive in recent decades (Fig. 1a, blue line).

Over southern Australia, ENSO and the IOD are both negatively correlated with SAR^17^,^18^, i.e., an El Niño and a positive IOD each alone can reduced rainfall. Somewhat similar variations in the relationship between ISMR and tropical variability occur: the SAR-ENSO and SAR-IOD relationships have changed substantially since the 1980s (Fig. 1b). Specifically, ENSO’s influence on SAR weakened (Fig. 1b, red line) while the IOD’s influence on SAR strengthened (Fig. 1b, blue line).

Although the cause of the changing ISMR-ENSO relationship has been a point of contention, the changing SAR-IOD relationship is part of the interlinked issue and has not been discussed previously. We propose that the level of ENSO-IOD coherence plays a role in the decadal variations of the relationship. ENSO-IOD coherence had been decreasing since the 1980s (Fig. 1a,b, black line), co-varying with the evolution of the ISMR-IOD and the SAR-ENSO correlation. Although these variations are demonstrated using reanalysed rainfall data, a similar result is found from rain-gauge based observations over India and Australia, highlighting the robustness of our result (Supplementary Fig. 1).

### Mechanism

On the basis of the time when the reversal in the ISMR-IOD relationship occurred, we choose two sub-periods to depict the associated anomalous circulation patterns. The two sub-periods are: 1966–1982 when the ISMR-IOD correlation is negative and ENSO-IOD coherence is high, and 1992–2008 when the ISMR-IOD correlation is positive and ENSO-IOD coherence is low (Fig. 1, grey boxes).

**Figure 2:**
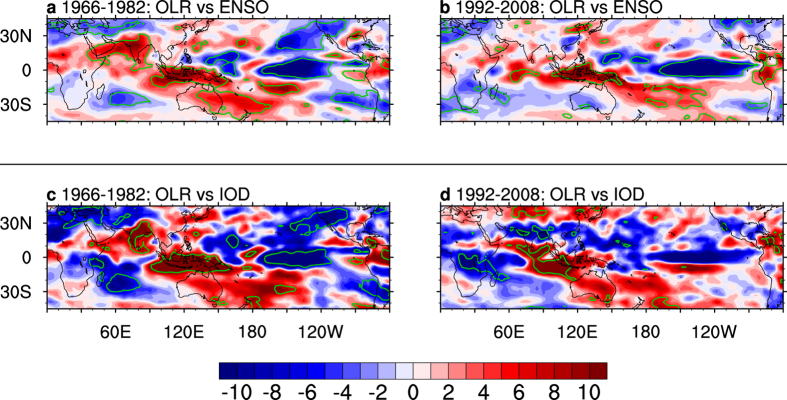
Regression maps of OLR anomalies (W m^−2^) onto ENSO and the IOD. (**a**) ENSO-induced, and **(c)** IOD-induced OLR variations during the sub-period 1966–1982, and (**b,d**) those of the sub-period 1992–2008. Anomalies are subtracted from the seasonal climatology of 1948–2010. Units are shown for a one standard deviation of the predictor in each panel. Bold green contours denote areas where the correlation is statistically significant above the 95% confidence level. The significance is calculated using a two-tailed Student’s *t* test. All maps were generated by NCL version 6.3.0 (http://dx.doi.org/10.5065/D6WD3XH5).

**Figure 3:**
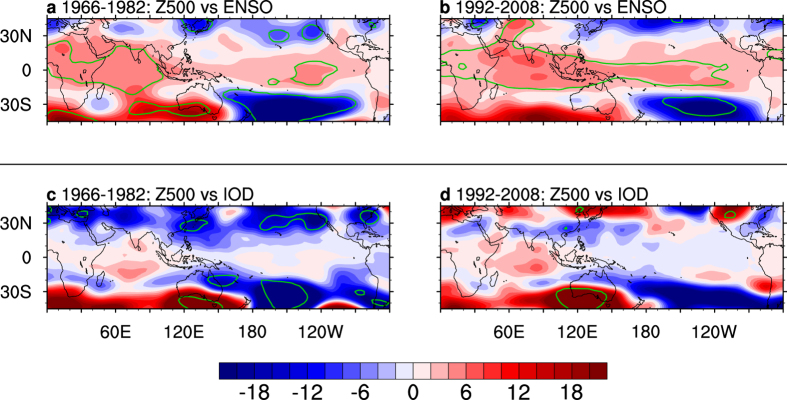
Regression maps of Z500 anomalies (m) onto ENSO and the IOD. (**a**) ENSO-induced, and (**c**) IOD-induced Z500 variations during the sub-period 1966–1982, and (**b,d**) those of the sub-period 1992–2008. Anomalies are subtracted from the seasonal climatology of 1948–2010. Units are shown for a one standard deviation of the predictor in each panel. Bold green contours denote areas where the correlation is statistically significant above the 95% confidence level. The significance is calculated using a two-tailed Student’s *t* test. All maps were generated by NCL version 6.3.0 (http://dx.doi.org/10.5065/D6WD3XH5).

In order to examine atmospheric circulation anomalies associated with the varying ENSO-IOD coherence, we regress grid-point outgoing longwave radiation (OLR, a proxy for convection, Fig. 2) and 500 hPa geopotential height (Z500, Fig. 3) onto indices of ENSO and the IOD for each of the two periods. During the strong coherence period of 1966–1982 (Fig. 2a,c), convection anomalies associated with ENSO are seen in the tropical eastern Indian Ocean and the tropical Pacific, reflecting the strong ENSO-IOD coherence. In particular, anomalies indicating suppressed convection are seen over the Indian subcontinent and over southeast Australia. These results are physically consistent with the finding that during an El Niño, anomalous subsidence and high mean sea level pressure (MSLP) are generated over the western-sphere of the tropics (Fig. 2a,b and Supplementary Fig. 2a,b), including the Indian subcontinent and eastern Australia, suppressing convection and rainfall^29^,^30^.

Convection anomalies over the Indian subcontinent and eastern Australia are absent during the weak ENSO-IOD coherence of 1992–2008 (Fig. 2b). During this period, anomalies associated with ENSO and the IOD are more mutually independent, as shown by a general lack of ENSO-induced anomalies in the tropical eastern Indian Ocean (Fig. 2b), or a lack of IOD-induced anomalies in the central/eastern tropical Pacific (Fig. 2d). A positive IOD induces enhanced convection in the vicinity of the Indian subcontinent (Fig. 2d), in sharp contrast to the suppressed convection during the high-coherence of 1966–1982 (Fig. 2c). As discussed in previous studies^15^,^16^, during a positive IOD event without ENSO’s dominance, colder than normal sea surface temperatures (SSTs) and enhanced subsidence in the eastern pole lead to meridional circulation anomalies featuring anomalous winds blowing north-westwards to the Indian subcontinent. The flow promotes ascending motion in the monsoon trough, favourable to rainfall in the Indian subcontinent.

Overall, ENSO and the IOD play an opposite role in ISMR variability and this in conjunction with the changing ENSO-IOD coherence explains the post-1980 weakening in the ISMR-ENSO relationship. During the high ENSO-IOD coherence of 1966–1982, El Niño-induced high MSLP anomalies over the Indian subcontinent are physically consistent with the anomalous subsidence, resulting in a reduction of rainfall (Fig. 2a and Supplementary Fig. 2a). Because of the strong pre-1980 ENSO-IOD coherence, the response of ISMR to ENSO dominates and offsets the opposing impact from the IOD, leading to a net anomalous subsidence and decreased rainfall associated with the positive IOD (Fig. 2c). By contrast, during 1992–2008, because of the weak ENSO-IOD coherence, the impact from ENSO no longer dominates (Fig. 2b). Instead, the influence of the IOD on ISMR, now without the offsetting effect of ENSO, manifests itself (Fig. 2d).

Likewise, the post-1980 breakdown of the SAR-ENSO relationship is at least partly caused by the post-1980 weakening of ENSO-IOD coherence, although the mechanism is somewhat different. SST anomalies associated with ENSO drive both tropical and extratropical responses. In addition to the baroclinic response to the anomalous convective heating, which leads to a hemispheric-wide, tropically trapped pattern resembling the Southern Oscillation^31^,^32^, the same diabatic heating also excites equivalent barotropic Rossby wave trains, such as the Pacific–South America pattern that emanates from the tropics to the extratropics^33^. Neither of these responses has a direct pathway to impact southern Australia^18^. The extratropical impacts of the IOD are conducted through similar pathways during austral winter and spring^18^. Diabatic heating anomalies from the west and east poles of the IOD generate Rossby wave trains, with a high pressure anomaly centre south of Australia during a positive IOD. There is no direct pathway for ENSO to influence SAR, because ENSO’s impact on SAR can only be delivered through reinforcing the IOD, via ENSO-IOD coherence^18^. Furthermore, it is the first time to examine variations in the relationship between SAR and tropical variability.

During the high ENSO-IOD coherence period of 1966–1982, as a result of the superimposing effect from ENSO and the IOD, convective anomalies in the eastern tropical Indian Ocean excite a strong Rossby wave train, which has a high geopotential height centre over southern Australia (Fig. 3a). This is far greater than that during the weak ENSO-IOD coherence of 1992–2008 (Fig. 3b). Stronger height anomalies due to the high coherence make greater easterly wind anomalies, which weaken and divert the rain-bearing eastward-moving weather systems^17^,^18^, leading to a greater rainfall reduction over southern Australia compared to the period of weak coherence.

Over the past two decades, the frequency of positive IOD has been increasing^7^. The increased frequency represents a strong signal-to-noise ratio over the period that maintains a high negative correlation seen in Fig. 1b (blue), despite the lack of coherent El Niño events. There is a commensurately stronger high pressure centre south of Australia facilitating the stronger impact (Compare Fig. 3c,d).

### Does climate change play a part?

That the post-1980 weakening in the ISMR-ENSO relationship is linked to a change in ENSO-IOD coherence has important implications for attributing whether greenhouse warming has played a part in the observed weakening. This is an issue that attracts much attention^21^,^23^. Although it is not a trivial issue to attribute the cause for observed changes, as this would require a systematic set of well thought-out experiments, one might be able to address whether under greenhouse warming occurrences of such weakening will change in frequency and whether ENSO-IOD coherence will undergo a significant change.

**Figure 4:**
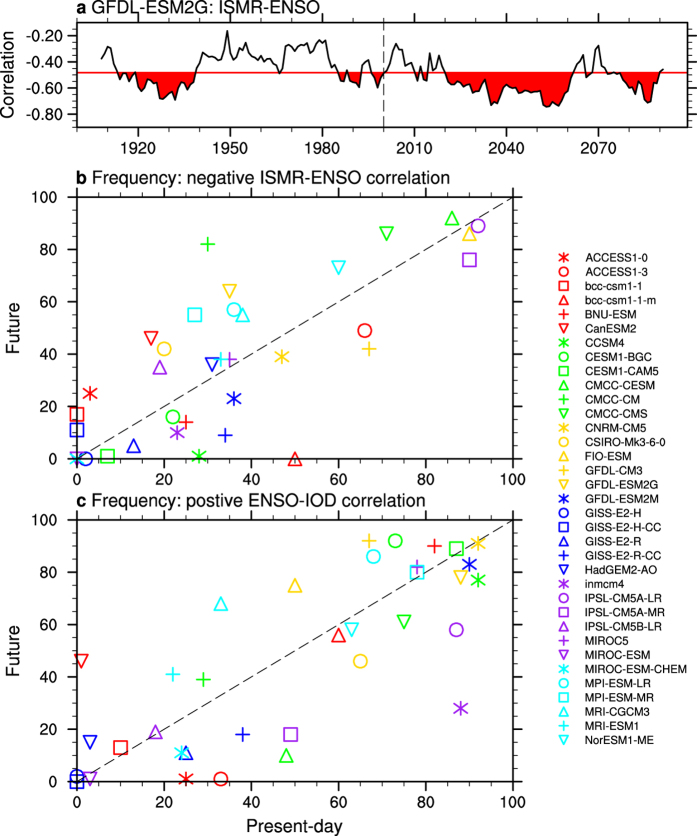
ISMR-ENSO and ENSO-IOD relationships in CMIP5 historical and RCP8.5 simulations. (**a**) Sliding correlation between ISMR and ENSO using 17-year sliding windows during a 200-year simulation (1900–2099) in GFDL-ESM2G. In this case, there are 35 occasions in the present-day and 64 cases in the future in which the sliding correlations are significant (red shaded regions). (**b,c**) Same method as (**a**) is carried out in all models for ISMR-ENSO and ENSO-IOD relationships, respectively. Dashed lines in (**b,c**) are diagonals, supporting there is no intermodel consensus on how the frequency of ISMR-ENSO weakening will change under greenhouse warming. All were generated by NCL version 6.3.0 (http://dx.doi.org/10.5065/D6WD3XH5).

We analysed output from 35 CMIP5 models, each run for a 200 years (1900–2099) using historical simulations before 2006 and Representative Concentration Pathway 8.5 (RCP8.5) simulations thereafter^28^. Detailed model information is provided in Supplementary Table 1. The GFDL-ESM2G model result is used as an illustrative example. A time series of 17-year running correlations is constructed, and divided into two periods: the first century as present-day and the second century as future (Fig. 4a). For each model, we count the number of occurrences in which a correlation is statistically significant for each 17-year period, i.e., when the ENSO impact is strong. In this case, there are 35 occasions in the present-day and 64 in the future in which the 17-year sliding window correlations are significant. This is carried out for all models and the results are recorded in Fig. 4b. There is no intermodel consensus on how the frequency of the ISMR-ENSO weakening will change, with 18 models showing a higher frequency of a statistically significant correlation in the future.

Consistently, there is no consensus on how the frequency of occurrences in which ENSO-IOD correlation reaches a statistically significant level in any 17-year window will change (Fig. 4c). Over the full 100-year periods of present-day and future climate, there is no consensus either (Supplementary Fig. 3). Therefore, we can conclude that there is no modelling evidence to suggest that under greenhouse warming, the occurrences of a weakening ISMR-ENSO relationship or breakdowns of ENSO-IOD coherence will occur more frequently. This points out the possibility that the post-1980 weakening of the ISMR-ENSO relationship is largely caused by internal climate variability. The same can be said about the post-1980 weakening in the SAR-ENSO relationship.

## Discussion

We have shown that changes in the impact of ENSO and IOD on regional rainfall before and after 1980 are partly attributable to varying ENSO-IOD coherence. For the Indian subcontinent, ENSO’s impact with an El Niño leading to deficit rainfall is opposite to that of a positive IOD, which without ENSO’s influence leads to increased rainfall during a positive IOD. As such, in the pre-1980 period when ENSO and the IOD are strongly coherent, ENSO dominates and overwhelms the IOD’s impact. Therefore, both an El Niño and a positive IOD are seen to be associated with reduced rainfall. In the post-1980 period when the coherence is weak, the rain-inducing impact from the IOD dominates.

For southern Australia, both an El Niño and a positive IOD lead to reduced rainfall and the impact of an El Niño is conducted by reinforcing a positive IOD, and vice versa. Therefore, in the pre-1980 period when ENSO-IOD coherence is high, ENSO’s influence appears greater. During the post-1980 period when ENSO-IOD coherence is weak, El Niño events do not occur concurrently with a positive IOD and there is no pathway for El Niño to influence southern Australia. This causes SAR to be dominated by positive IODs.

ENSO-IOD coherence allows us to evaluate whether these observed variations are attributable to greenhouse warming. There is no intermodel consensus on how the coherence will change. This suggests that the likelihood of greenhouse warming playing a key role is small, although we note that an ultimate attribution requires a set of systematic model experiments with and without climate change forcing.

There are many avenues whereby the present study can be extended. One such avenue is the role of internal variability in forcing fluctuations of the ENSO-IOD coherence. For example, there are more frequent occurrences of ENSO Modoki since 1980s^34^, and the extent to which this is a potential forcing for the varying ENSO-IOD coherence need to be investigated, although there is no relationship between ENSO Modoki and the IOD during these two sub-periods. Further, the centre of convective activities associated with El Niño and La Niña is quite different, and the associated teleconnection to regional climate in the two manifestations of the ENSO-IOD coherence, i.e., La Niña with a concurrent negative IOD and El Niño with a concurrent positive IOD, may not be symmetric. Further, there are other tropical modes that can concurrently influence rainfall in different regions and modulate teleconnections on interdecadal timescales^35^. A detailed examination of such issues is needed.

## Data and Methods

### Data

For observations, HadISST monthly sea surface temperatures^36^ are used to construct ENSO and IOD indices. Monthly rainfall from the NCEP/NCAR Reanalysis^37^ is used to construct regional rainfall indices, including the ISMR and SAR. MSLP, Z500 and OLR from NCEP/NCAR Reanalysis are used to analyse responses of atmospheric circulations to tropical variability. All observed time series and grid-point data are quadratically detrended to remove the impact of a long-term trend. For models, we use SST and rainfall from both the historical and RCP8.5 simulations of 35 CMIP5 models. All variables are detrended quadratically over the 200-year period to remove long-term trends.

### Methods

We employ two methods to define tropical variability indices depending on the characteristic of the data. In observations, the IOD is defined using the Dipole Mode Index (DMI), the difference of the area-averaged SST anomalies in the west IOD pole (10°S–10°N, 50°E–70°E) and the east pole (10°S-Eq., 90°E–110°E)^6^. ENSO is defined using the Niño3 index; area-averaged SST anomalies in the Niño3 region (5°S–5°N, 90°W–150°W). For models, because coupled models have their own variability, we use empirical orthogonal function (EOF) analysis to define tropical variability^38^. The IOD is described through EOF analysis on SST anomalies in the tropical Indian Ocean domain (25°S–25°N, 40°E–120°E) and the IOD index is represented by the time series associated with the spatial pattern of the IOD mode. ENSO is also represented by the time series associated with the spatial pattern of the ENSO mode through EOF analysis on SST anomalies in the tropical Pacific domain (25°S–25°N, 120°E–80°W). Time series of ISMR and SAR are constructed by area-averaged rainfall anomalies over the Indian subcontinent (70°E–88°E, 7°N–28°N) and southern Australia (142°E–147°E, 37°S–40°S), respectively. Linear regression and correlation with/without a 17-year sliding window are used in this study to examine inter-decadal variations between tropical variability and regional rainfall.

### Seasonality

All calculations in this paper are based on rainfall over the Indian subcontinent and southern Australia in the main rainfall season (June, July, August and September).

### Graphic software

All plots and maps were generated by NCL version 6.3.0 http://dx.doi.org/10.5065/D6WD3XH5.

## Additional Information

**How to cite this article**: Li, Z. *et al*. Dynamics of changing impacts of tropical Indo-Pacific variability on Indian and Australian rainfall. *Sci. Rep.* **6**, 31767; doi: 10.1038/srep31767 (2016).

## Supplementary Material

Supplementary Information
