# Supplementary material for: Dynamics of changing impacts of tropical Indo-Pacific variability on Indian and Australian rainfall
Source: Sci Rep. 2016 Aug 22;6:31767. doi: 10.1038/srep31767 (PMC4992885; doi:10.1038/srep31767)

# Dynamics of changing impacts of tropical Indo-Pacific variability on Indian and Australian rainfall

Ziguang Li^1, 2^, Wenju Cai^2, 1^ and Xiaopei Lin^1^*

^1^ Physical Oceanography Laboratory/CIMST, Ocean University of China and Qingdao National Laboratory for Marine Science and Technology, Qingdao 266100, China.

^2^ CSIRO Oceans and Atmosphere, Aspendale, VIC 3195, Australia.

**Supplementary Figures**

We reproduce Fig.1, using two rain-gauge based observational rainfall datasets over India^1^ and Australia^2^, to demonstrate the robustness of our result from NCEP/NCAR Reanalysis dataset.


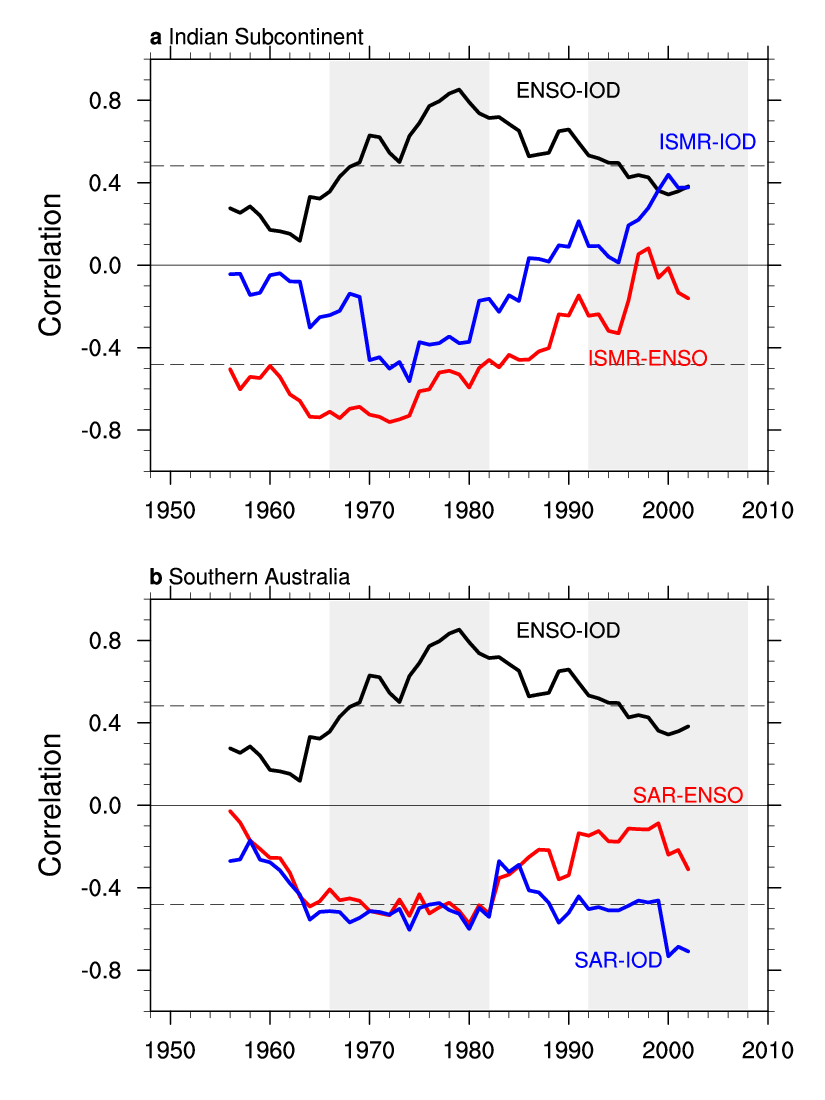


**Figure S1 | Sliding correlation between tropical variability and regional rainfall. (a)** Sliding correlations between JJAS seasonal anomalies of the ISMR and ENSO (**red** line), the ISMR and the IOD (**blue** line), and ENSO and IOD (**black** line) on 17-year sliding windows. Values are plotted at the centre of each 17-year period and statistical significance above the 95% confidence level by a two- tailed Student’s *t*-test is achieved when the correlation is greater than 0.482 in amplitude, which indicated by dashed lines. Shaded boxes denote the sub-periods of 1966-1982 and 1992-2008, respectively. **(b)** As in **(a)**, but for SAR. All plots were generated by NCL version 6.3.0 (http://dx.doi.org/10.5065/D6WD3XH5).

**References**

1. Parthasarathy, B., Munot, A.A. and Kothawale, D.R. All-India monthly and seasonal rainfall series: 1871–1993. *Theoretical and Applied Climatology*,*49*(4), pp.217-224 (1994).
2. Jones, D.A., Wang, W. and Fawcett, R. High-quality spatial climate data-sets for Australia. *Australian Meteorological and Oceanographic Journal*,*58*(4), p.233 (2009).


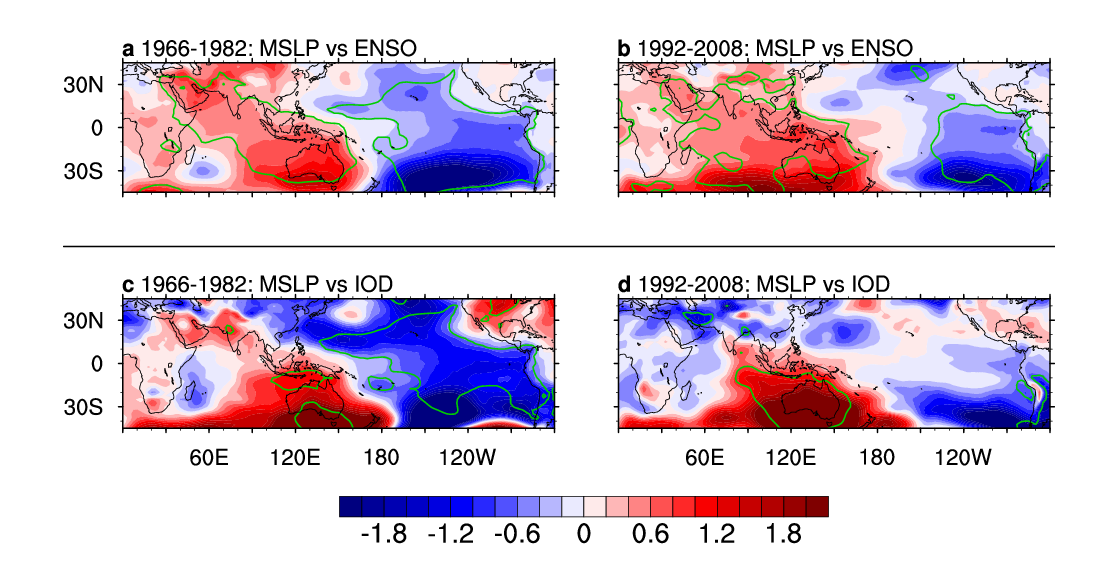


**Figure S2 | Regression maps of MSLP anomalies (hPa) onto ENSO and the IOD.** **(a)** ENSO-induced, and **(c)** IOD-induced MSLP variations during the sub-period 1966-1982, and **(b)** and **(d)** those of the sub-period 1992-2008. Anomalies are subtracted from the seasonal climatology of 1948-2010. Units are shown for a one standard deviation of the predictor in each panel. Bold green contours denote areas where the correlation is statistically significant above the 95% confidence level. The significance is calculated using a two-tailed Student’s *t*-test. All maps were generated by NCL version 6.3.0 (http://dx.doi.org/10.5065/D6WD3XH5).


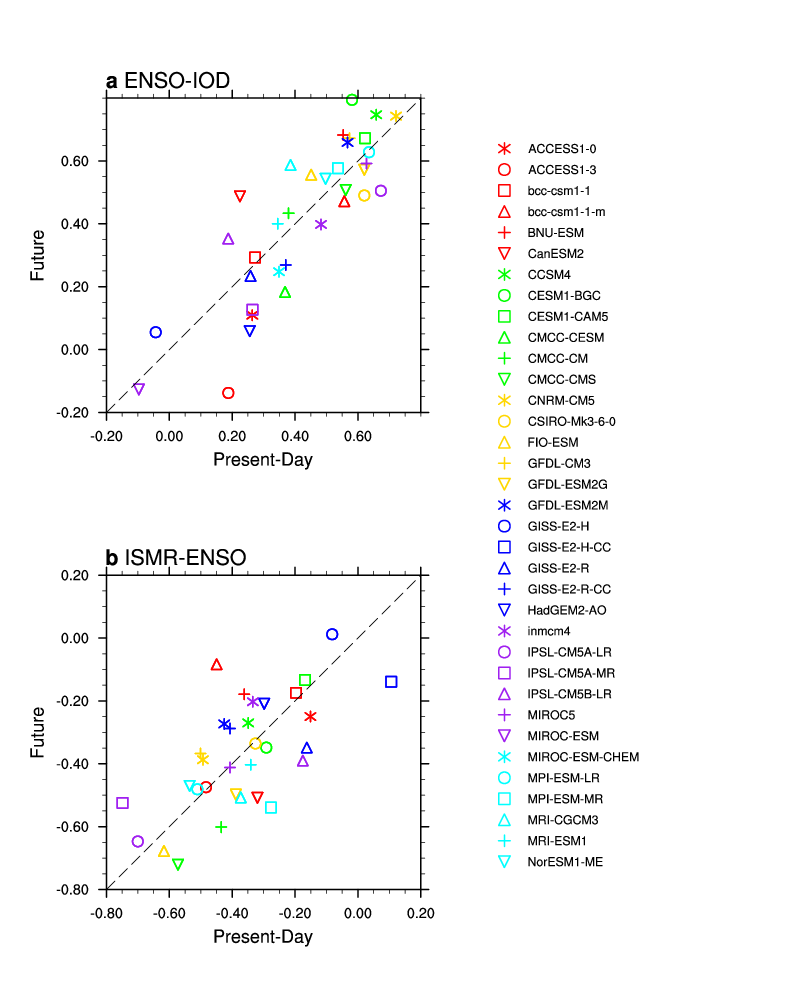


**Figure S3 | ISMR-ENSO and ENSO-IOD relationships in CMIP5 historical and RCP8.5 simulations. (a)** Correlation between ISMR and ENSO over the full 100 years of the present-day and future climate. **(b),** as in **(a)**, but for the correlation between ENSO and IOD. Dashed lines in **(a)** and **(b)** represent no changes. Models are distributed around the dashed lines supporting that there is no intermodel consensus for future climate change. All plots were generated by NCL version 6.3.0 (http://dx.doi.org/10.5065/D6WD3XH5).

**SupplementaryTable**

**Table S1 | Detailed information of CMIP5 models, including modelling centre or group, institute ID and model name.**


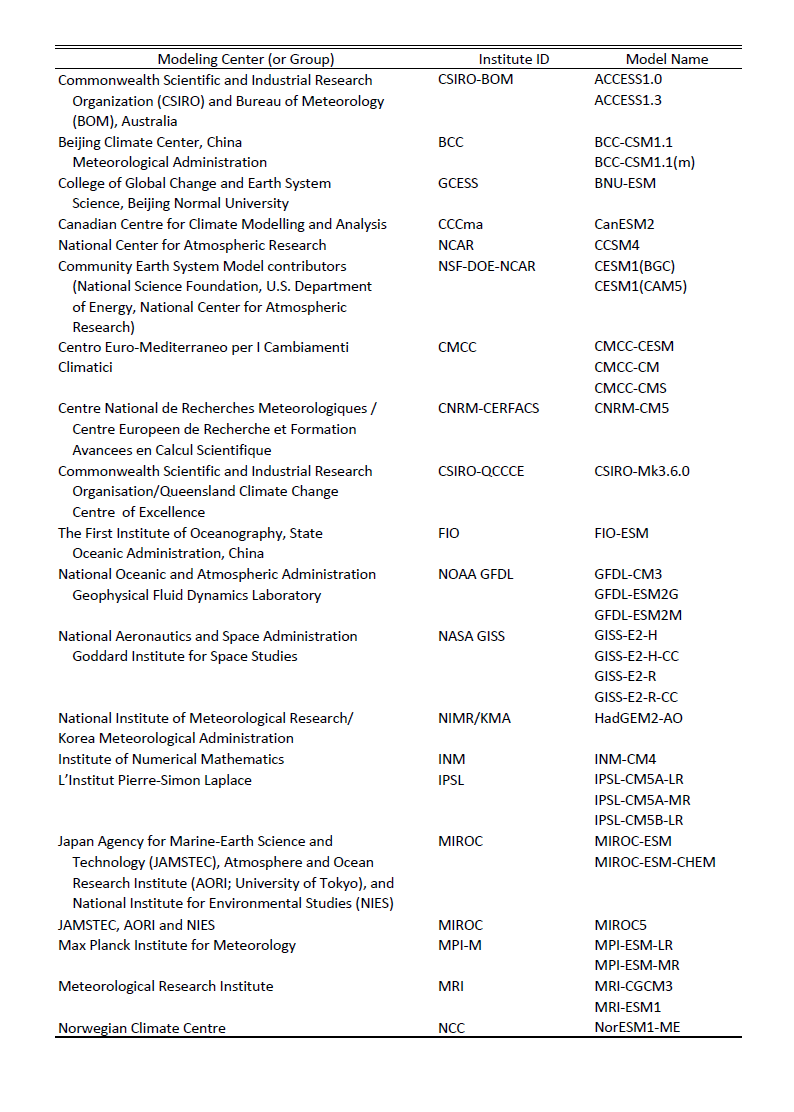

Supplement: Supplementary Information [file srep31767-s1.doc]
